# JNK activation in TA and EDL muscle is load-dependent in rats receiving identical excitation patterns

**DOI:** 10.1038/s41598-021-94930-x

**Published:** 2021-08-12

**Authors:** Einar Eftestøl, Martino V. Franchi, Stephanie Kasper, Martin Flück

**Affiliations:** 1 Department of Biosciences, University of Oslo, Kristine Bonnevies hus, Blindernveien 31, 0371 Oslo, Norway; 2 Laboratory for Muscle Plasticity, Department of Orthopaedics, University of Zürich, Zurich, Switzerland; 3 Department of Biomedical Sciences, University of Padova, Padua, Italy

**Keywords:** Animal physiology, Calcium signalling, Stress signalling, Phosphorylation

## Abstract

As the excitation–contraction coupling is inseparable during voluntary exercise, the relative contribution of the mechanical and neural input on hypertrophy-related molecular signalling is still poorly understood. Herein, we use a rat in-vivo strength exercise model with an electrically-induced standardized excitation pattern, previously shown to induce a load-dependent increase in myonuclear number and hypertrophy, to study acute effects of load on molecular signalling. We assessed protein abundance and specific phosphorylation of the four protein kinases FAK, mTOR, p70S6K and JNK after 2, 10 and 28 min of a low- or high-load contraction, in order to assess the effects of load, exercise duration and muscle-type on their response to exercise. Specific phosphorylation of mTOR, p70S6K and JNK was increased after 28 min of exercise under the low- and high-load protocol. Elevated phosphorylation of mTOR and JNK was detectable already after 2 and 10 min of exercise, respectively, but greatest after 28 min of exercise, and JNK phosphorylation was highly load-dependent. The abundance of all four kinases was higher in TA compared to EDL muscle, p70S6K abundance was increased after exercise in a load-independent manner, and FAK and JNK abundance was reduced after 28 min of exercise in both the exercised and control muscles. In conclusion, the current study shows that JNK activation after a single resistance exercise is load-specific, resembling the previously reported degree of myonuclear accrual and muscle hypertrophy with repetition of the exercise stimulus.

## Introduction

The biomechanical stress produced in a contracting muscle is believed to activate biochemical^1^ and biomechanical^2^ signalling pathways, termed mechanotransduction^3,4^, leading to increased biosynthetic efficiency and capacity that in the long-run result in functional muscle growth^5^. A de novo hypertrophic response is also preceded^6^ and determined^7^ by myonuclear accretion^8,9^, further contributing to increased synthetic capacity.

Although there is scarce experimental evidence for the underlying mechanisms, biomechanical stress is widely accepted as a primary trigger for muscle hypertrophy^10–12^, and the effect of e.g. voluntary strength exercise or synergist-ablation induced overload on immediate-early signalling cues involved in protein synthesis and/or the long-term hypertrophic response is, by and large, implicated in mechanotransduction^13^. However, under the above-mentioned conditions, as well as under most other experimental conditions, the neural input changes concomitantly, as an increased firing frequency and motor unit recruitment orchestrates the magnitude of force output and the following biomechanical stress^14–19^. Thus, under experimental conditions that do not separately control neural input and mechanical stimuli it is difficult to properly address the importance of mechanotransduction in hypertrophy; it follows that the intrinsic nature of the molecular pathways that are implicated in the mechano-induced activation of muscle protein synthesis is still poorly characterized and understood^20–22^.

A well characterized route of activation of skeletal muscle protein synthesis (and subsequent hypertrophy) in response to resistance exercise is regulated by changes in the translational efficiency and capacity via signalling through coupled kinases, and is further increased through myonuclear accrual^23–26^. The increased translational capacity following resistance exercise in humans is correlated with increased muscle mass^27–29^. The non-receptor tyrosine kinase FAK (focal adhesion kinase), and the downstream serine/threonine phosphotransferases mTOR (mammalian target of rapamycin) and p70S6K (70 kDa ribosomal protein S6 kinase) are implicated in the load-induced hypertrophic response in cell culture^30^ and after reloading of unloaded rats^31^. Further, the post-translational modification and subsequent activation of FAK and the functionally-associated mixed-lineage kinase JNK (c-jun N-terminal kinase), as well as mTOR and p70S6K, correlate with the mechanical load impact during acute electrically-paced exercise^32–34^. FAK and JNK have further been regarded to take part in the load-regulated myogenic adaptations in skeletal muscle^35,36^. Especially, we have previously found that quantitative increases in the abundance and Y397 phosphorylation of FAK act upstream of muscle loading-related phosphorylation of T183/Y185-JNK and downstream of S63-c-jun and T421/S424-p70S6K^31,37^. Interestingly, these mechanical pathways are also suggested to act synergistically with changes in amino acid uptake and growth factor signalling to sustain mTOR and FAK signalling^21,38,39^. Thus, signalling pathways activated by e.g. mechano-, nutrient- and growth factor-stimuli probably modulate each other rather than working as independent pathways.

A few experimental studies have to a certain extent specifically addressed acute^32–35,40,41^ or long-term^34,42^ effects of mechanical stimuli per se, either by passively stretching innervated^33^, (functionally) denervated^43–45^ or isolated^35^ muscles, or by standardizing the neural input by electrically-paced contractions either in vivo^32–34,40–42,46^ or in isolated muscles^47,48^. However, a more comprehensive mapping and understanding of the activation of early mechanotransduction pathways connected to long-term outcomes are still warranted. To start on this quest, we previously developed a rat strength-exercise model and found that after a 6-week exercise regime under identical neural activity, differences in mechanical conditions had a major effect on the number of myonuclei and fibre size but not on fibre type^22^. Also, after one acute session a few molecular correlates were indicated in the mechano-response, e.g. myogenin and MRF4, implicated both in satellite cell activation and increased transcriptional response. Yet, no significant effect on the previously proposed mechano-responsive kinases Akt and p70S6K was observed^22^.

Thus, herein we investigate in more detail the acute responses to a single bout of the previously characterized exercise-model^22^ related to the proposed mechanosensitive kinases FAK, mTOR, p70S6K and JNK. We find that JNK signalling is highly sensitive to mechanical stimuli, and propose a potential role for all 4 kinases in the following mechano-dependent hypertrophic response.

## Results

A single exercise session of electrically-paced contractions was evaluated for effects on specific phosphorylation (phosphorylated protein / total protein abundance of the respective protein) as well as total protein abundance (per 33 µg of isolated muscle protein) for the kinases FAK, mTOR, p70S6K and JNK. 50 rats were unilaterally exercised for a total time of 2 (N = 16), 10 (N = 18) or 28 (N = 16) minutes by stimulating the dorsiflexor muscle group (TA and EDL) non-invasively through skin-electrodes. Groups were equally allocated between a high-load or a low-load contraction, contralateral muscles functioning as non-stimulated controls. The low-load group obtained a peak tension of 50–60% of the high-load group.

### Effects of a single high- or low-load exercise bout on the phosphorylation of FAK, mTOR, p70S6K and JNK in the EDL and TA muscle

**Figure 1:**
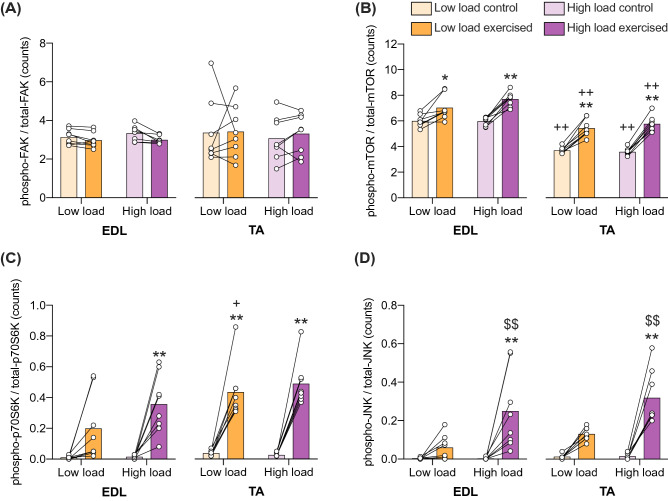
Exercise-induced JNK phosphorylation is load-dependent, while mTOR and p70S6K phosphorylation is muscle-type dependent. Specific phosphorylation (phosphorylated protein / total protein) of Y397-FAK (**a**), pS2448-mTOR (**b**), pT421/S424-p70S6K (**c**), and pT183/Y185-JNK (**d**) as measured in 33 µg soluble protein from exercised and contralateral control TA and EDL muscles after a 28-min exercise stimulus with a high or a low load. Post-hoc significant differences denoted with *, ** p < 0.05 and 0.01 versus control leg; + ,  ++ p < 0.05 and 0.01 vs EDL within same exercise group; $$ p < 0.01 versus low load exercised group within the same muscle. Data are presented as individual muscle values (circles), contralateral comparisons (lines) and group means (bars) (n = 8 per group).

A single 28-min-long exercise stimulus led to an increased specific phosphorylation of S2448-mTOR, T421/S424-p70S6K, and T183/Y185-JNK, but not Y397-FAK, in both the TA and EDL muscle compared to contralateral non-exercised controls (ANOVA results, see Table 1). At the post-hoc level, there was an effect of exercise in the high-load group in both EDL and TA for mTOR, p70S6K and JNK, but only for mTOR (EDL and TA) and p70S6K (TA) in the low-load group (Fig. 1). In addition, JNK had an increased specific phosphorylation in the exercised leg after the high-load compared to the low-load exercise for both EDL (p = 0.0085) and TA (p = 0.0097) (Fig. 1d). No such load-effect was observed at the post-hoc level for mTOR (Fig. 1b) or p70S6K (Fig. 1c). Total abundance (count) of phosphorylated protein per 33 µg of isolated muscle protein (not relative to respective kinase abundance) is shown in Supplementary Figures 1 and 2.

**Table 1:** Three-way ANOVA for the factors exercise, load and muscle type (corresponding to Figs. 1 and 2), or exercise, load and exercise duration (corresponding to Figs. 3 and 4), showing percent of total variation and corresponding p-value for the respective factors and interactions.

Figure 1	p-FAK		p-mTOR		p-p70S6K		p-JNK	
Source of variation	%	P value	%	P value	%	P value	%	P value
Load	0.556	0.4259	0.5599	0.2334	1.321	**0.0094**	10.82	**0.0016**
Muscle	2.355	0.5644	50.33	**<** **0.0001**	5.15	**0.0154**	2.055	0.1333
Exercise	0.0686	0.8325	33.62	**<** **0.0001**	58.32	**<** **0.0001**	39.85	**0.0001**
Load × muscle	0.0686	0.7769	0.1308	0.528	0.444	0.471	0.00001881	0.9959
Load × exercise	0.2809	0.5213	1.013	**0.0249**	1.398	**0.0041**	10.64	**0.0015**
Muscle × exercise	0.208	0.511	1.009	**0.0227**	3.289	**0.0192**	1.075	0.2722
Load × muscle × exercise	0.003427	0.9639	0.0448	0.4715	0.2354	0.5938	0.002939	0.9467

Figure 2	FAK		mTOR		p70S6K		JNK	
Source of variation	%	P value	%	P value	%	P value	%	P value
Load	0.07586	0.5918	0.0011	0.9107	0.0002	0.9746	0.6114	**0.0282**
Muscle	60.22	**0.0015**	95.81	**<** ** 0.0001**	87	**<** ** 0.0001**	66.89	**0.0004**
Exercise	0.7377	0.2619	0.1215	0.1679	3.463	**<** ** 0.0001**	3.744	**0.0323**
Load × muscle	0.01537	0.8323	0.0875	0.3562	0.1765	0.3254	0.9	**0.0277**
Load × exercise	0.4963	0.0956	0.0311	0.4017	0.0129	0.7796	1.483	**0.0154**
Muscle × exercise	1.093	**0.0286**	0.0572	0.306	1.449	**0.0058**	0.4823	0.283
Load × muscle × exercise	0.2397	0.4947	0.0399	0.3746	0.0112	0.7859	0.003449	0.8932

Figure 3	p-FAK		p-mTOR		p-JNK			
Source of variation	%	P value	%	P value	%	P value		
Duration	2.946	0.4101	30.66	**< 0.0001**	20.39	**< 0.0001**		
Load	1.03	0.1746	6.028	**0.0045**	3.367	**0.0022**		
Exercise	0.4182	0.5661	29.12	**< 0.0001**	25.87	**< 0.0001**		
Duration × load	0.0264	0.9714	2.134	0.1577	4.707	**0.0022**		
Duration × exercise	1.462	0.1197	2.253	**0.0003**	18.73	**< 0.0001**		
Load × exercise	0.006945	0.9117	0.01774	0.7578	2.999	**0.0023**		
Duration × load × exercise	0.116	0.9316	0.5369	0.1928	4.647	**0.0006**		

Figure 3	FAK		mTOR		JNK			
Source of variation	%	P value	%	P value	%	P value		
Duration	41.37	**0.0014**	10.92	0.0634	25.56	**0.0008**		
Load	0.02836	0.857	1.044	0.4124	2.487	0.0875		
Exercise	0.6267	0.068	1.644	0.0595	0.093	0.5708		
Duration × load	0.04421	0.9728	2.138	0.1661	1.012	0.5435		
Duration × exercise	0.4248	0.3948	1.335	0.4997	1.916	0.2522		
Load × exercise	0.1771	0.3908	0.1599	0.3419	0.7331	0.2183		
Duration × load × exercise	0.1934	0.7647	0.4274	0.7462	0.2533	0.6828		

P values < 0.05 highlighted in bold.

### Muscle-type differences in specific kinase phosphorylation

The specific phosphorylation of S2448-mTOR (p < 0.001) and T421/S424-p70S6K (p = 0.015) differed between the two muscles tested (Table 1), being higher for all groups in EDL when compared to their corresponding TA muscles from the same leg for mTOR (Fig. 1b), and lower in EDL than TA for the low-load exercised group for p70S6K (Fig. 1c).

### Muscle-type-, exercise- and load-effects on kinase abundance

**Figure 2:**
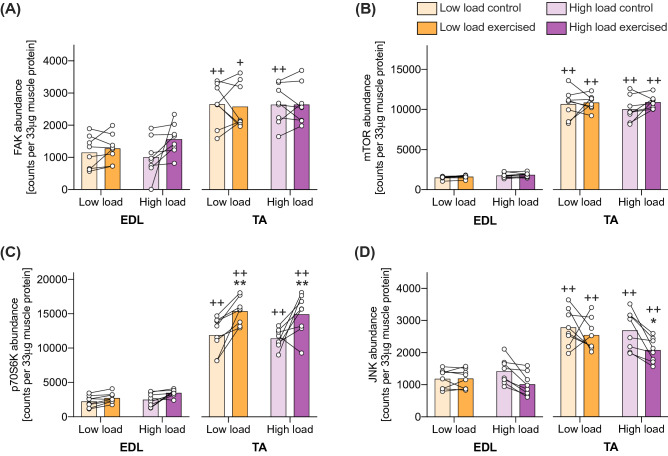
Kinase abundance is dependent on muscle type, and p70S6K abundance is increased by exercise in TA, while JNK abundance is decreased by exercise in a load-dependent manner in TA. Kinase abundance of FAK (A), mTOR (B), p70S6K (C), and JNK (D) as measured in 33 µg soluble protein from exercised and contralateral control TA and EDL muscles after a 28-min exercise stimulus with a high or a low load. Post-hoc significant differences denoted with *, ** p < 0.05 and 0.01 vs control leg; ++ p < 0.01 vs EDL within same exercise group. Data are presented as individual muscle values (circles), contralateral comparisons (lines) and group means (bars) (n = 8 per group).

The protein abundance of all assessed protein kinases was higher in TA compared to EDL (Table 1). FAK and JNK protein abundance was ~ 2-times higher, and mTOR and p70S6K abundance was ~ 6-times higher in TA compared to EDL muscles (Fig. 2).

There was an increased abundance of p70S6K in exercised compared to control muscles for both low load (p = 0.007) and high load (p = 0.007) in TA, but not in EDL (Fig. 2c).

The abundance of JNK was reduced after 28 min of high-load exercise in TA (p = 0.041), but not after low-load exercise (Fig. 2d).

### Effects of exercise duration on the specific kinase phosphorylation in TA muscle

We further assessed whether the increase in the specific phosphorylation of mTOR, JNK (and FAK) in TA muscle after 28 min of exercise would be evident also at earlier time-points, i.e., after 2 or 10 min of exercise, and whether it would be load-dependent. The specific phosphorylation of S2448-mTOR and T183/Y185-JNK, but not Y397-FAK, was affected by exercise in an exercise-duration dependent manner. The specific phosphorylation of T183/Y185-JNK, alone, was in addition dependent on load (Table 1).

**Figure 3:**
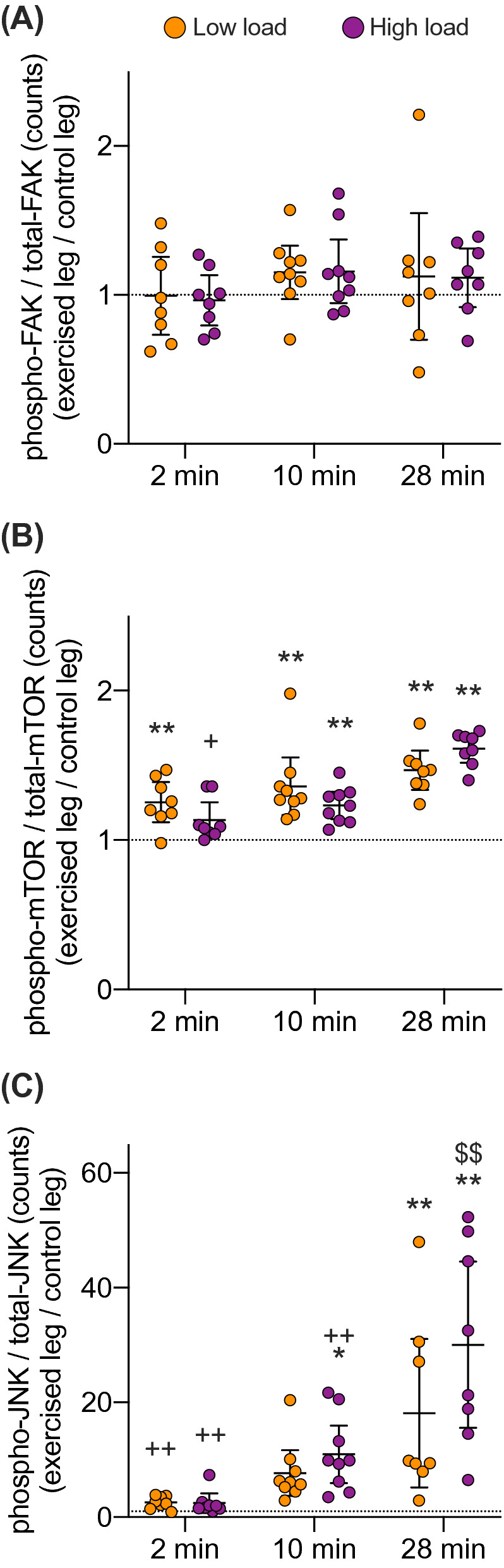
mTOR and JNK phosphorylation is increased with exercise duration, and for JNK also in a load-dependent manner. Specific phosphorylation of Y397-FAK (**a**), pS2448-mTOR (**b**) and pT183/Y185-JNK (**c**) shown as fold change in exercised relative to their respective contralateral control TA muscles after 2, 10 and 28 min of exercise with a high or a low load. Post-hoc significant differences denoted with *,** p < 0.05 and 0.01 versus control leg; + ,  ++ p < 0.05 and 0.01 versus 28 min exercise within loading groups; $$ p < 0.01 versus low load exercised group after 28 min of exercise. Data are presented as individual muscle values (circles) and group means with 95% CI (n = 8 per group for the 2 min and 28 min protocol, n = 9 per group for the 10 min protocol). Dotted line at y = 1 representing no difference between exercised leg and contralateral control leg.

At the post-hoc level, the specific phosphorylation of S2448-mTOR in TA muscle was increased in the exercised compared to the control leg after all durations of exercise, i.e., after 2, 10 and 28 min, in both the low-load and high-load group (for all comparisons p < 0.001) except for after 2 min in the high-load group (Fig. 3b). Specific phosphorylation of T183/Y185-JNK in TA muscle was increased in the exercised compared to the control leg after 10 (p = 0.028) and 28 min (p < 0.0001) of exercise in the high-load group, but only after 28 min in the low-load group (p < 0.0002) (Fig. 3c).

The specific phosphorylation of S2448-mTOR was increased after 28 min relative to 2 min of high-load (p = 0.016), but not low-load exercise (Fig. 3b). For T183/Y185-JNK, specific phosphorylation was increased after 28 min relative to 2 and 10 min of high-load (both p < 0.0001), but only relative to 2 min of low-load (p = 0.0007) exercise (Fig. 3c). Importantly, specific phosphorylation of T183/Y185-JNK was also increased in the high-load vs low-load group after 28 min of exercise (p < 0.0001), but not after 2- or 10 min (Fig. 3c). Total abundance (count) of phosphorylated protein per 33 µg of isolated muscle protein (not relative to respective kinase abundance) after 2-, 10- and 28 min of exercise is shown in Supplementary Figure 2.

### Effect of exercise duration on kinase abundance in TA muscle

**Figure 4:**
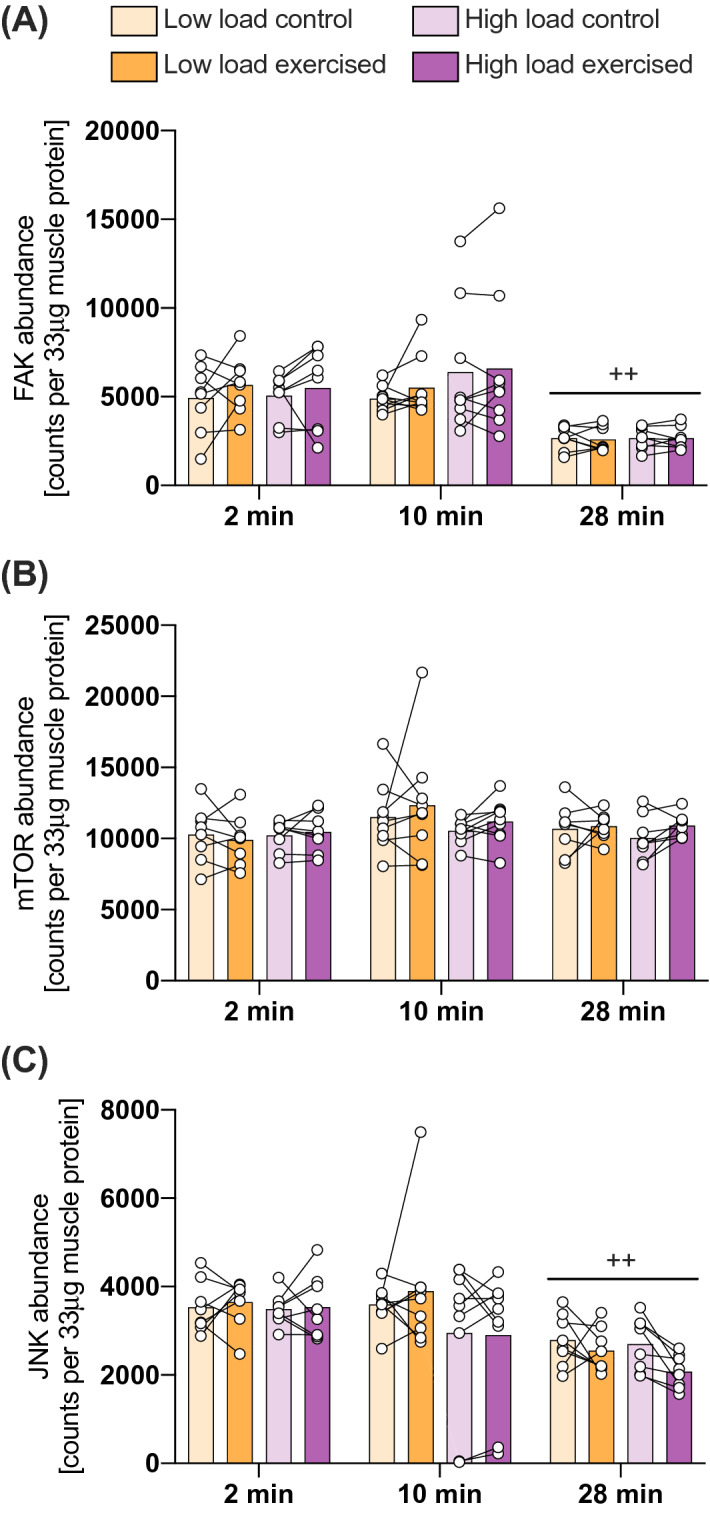
FAK and JNK abundance is decreased with exercise duration in both exercised and contralateral control leg, but not by exercise nor load per se. Kinase abundance of FAK (**a**), mTOR (**b**) and JNK (**c**) as measured in 33 µg soluble protein from exercised and contralateral control TA muscles after 2, 10 and 28 min of exercise with a high or a low load. Post-hoc significant differences denoted with ++ p < 0.01 versus 2- and 10-min exercise irrespective of group. Data are presented as individual muscle values (circles), contralateral comparisons (lines) and group means (bars) (n = 8 per group for the 2 min and 28 min protocol, n = 9 per group for the 10 min protocol).

We further assessed whether alterations in protein abundance contributed to the observed effects of exercise on the specific kinase phosphorylation in TA muscle. No effect of exercise nor load was observed for any of the kinases, but a main effect of exercise duration was identified for the abundance of FAK and JNK (Table 1). Compared to the 2- and 10-min duration of exercise, the abundance of FAK (p < 0.004) and JNK (p < 0.003) in TA muscle was lower after 28-min of exercise (Fig. 4).

## Discussion

The relationship between mechanical signalling following a single exercise stimulus and long-term adaptations under conditions where the neuronal input is standardized is poorly characterized. We here used an electrically-paced, in-vivo rat strength exercise model which has previously been shown to induce myonuclear accretion and hypertrophy^22^ in order to investigate early effects of mechanical load on the activation of FAK, mTOR, p70S6K and JNK. These kinases have been implicated in the pre-translational regulation of protein synthesis, and have previously been suggested to be involved in mechanotransduction. We show here that JNK activation is highly load-dependent, and propose that observed effects of exercise, load, exercise duration and muscle-type on JNK, mTOR and p70S6K phosphorylation and abundance is important for consolidating the hypertrophic response.

### JNK is activated by mechanical stimuli per se

We found JNK to be phosphorylated and thereby activated in a load-dependent manner in both TA and EDL. This is supported by an exercise-duration dependent effect already after 10 min of exercise only in the high-load group, increasing further after 28 min, and finally having increased JNK phosphorylation in the high-load group compared to the low-load group after the full 28-min exercise session. An interaction effect of exercise and load was also evident for mTOR and p70S6K. To our knowledge, the present result, for the first time, connect specific acute effects of load on JNK activation with established long-term hypertrophic effects of the same exercise stimulus^22^.

In rat plantaris muscle, an increased JNK phosphorylation has been shown to correlate with mechanical stress after a single bout of in situ eccentric (~ 10% length change) compared to concentric and isometric electrically-paced contractions^33^. JNK activation is further shown to be required in synergist ablation-induced muscle hypertrophy^35^, involving the counteraction of myostatin signals which inhibit the AKT-mTOR pathways and the upregulation of ubiquitin-mediated proteasomal degradation^49^. These effects seem also to involve the activation of Jun expression which is elevated by muscle stretch^50^ in interstitial nuclei (e.g., satellite cells) and myonuclei^51^ that may upregulate the promoter activity of muscle genes via Jun/Fos-heterodimers (e.g. AP-1)^52,53^. Nevertheless, the observed exercise-induced specific phosphorylation of JNK is likely not alone sufficient to explain the muscle- and load-dependent degree of hypertrophy observed in a previous study by Eftestøl et al.^22^ after the repeated impact of an identical 28-min protocol as herein over a period of 6 weeks. For instance, larger increases in the cross-sectional area of muscle fibres were reported with the high-load compared to the low-load protocol in the TA muscle (i.e., 33% vs 18%) compared to the EDL muscle (i.e., 16% vs 0%)^22^. The consistently higher abundance of all assessed protein kinases in TA compared to EDL combined with the muscle-type specific effects on mTOR and p70S6K phosphorylation observed herein reflects the previously observed higher degree of hypertrophy in TA compared to EDL muscle^22^. This correspondence suggests that quantitative differences in the expression of mTOR and p70S6K are part of the mechanism that sets the overall pace of the hypertrophic response. In addition, JNK activation could be related to other aspects of the observed load-dependent muscle responses such as the increased myonuclear number (i.e., satellite cell activation), as well as increased transcript expression of myogenin and MRF4 (myogenic regulatory factor 4)^22^.

Interestingly we observed an increased activation of both mTOR and p70S6K with exercise, including an interaction effect with load, yet this effect did not result at the level of statistical significance due to load per se. An important aspect of the present in-vivo setup is that due to the physical limits of the joint excursion-range, the peak tension in the low-load group could not be decreased to less than about 50–60% of the corresponding high-load group. This would require an even faster concentric contraction, resulting in a high-load isometric contraction towards the end of the stimulation. Notably, after 28 min of exercise, there was a consistently higher degree of phosphorylation (~ 10%) in the high-load vs low-load group for mTOR and p70S6K in both TA and EDL, which is also in line with previous observations for Akt and p70S6K phosphorylation^22^. It is thus intriguing to speculate that with a larger difference between the loading groups, all these kinases, that are widely known to be important in regulating protein synthesis (see introduction), would reveal a load-dependent activation. In fact, Rindom et al.^47^ has recently shown that, in response to acute exercise by electrically stimulating isolated rat EDL muscles, mTOR and p70S6K phosphorylation was load-dependent, while Jun and Fos mRNA transcription was dependent on both excitation and tension development^47^. Importantly, such in vitro setups are not prone to the same limitations as in-vivo setups, as they for instance are not limited by the joint excursion-range. Thus, through the use of chemical inhibitors of the muscle contraction together with passive stretch, Rindom et al.^47^ were able to compare a high-load active contraction to both a high-load passively stretched group with comparable tension development either with or without excitation, and also to an excited “low-load” group with ~ 100% reduction in tension development.

In summary, in light of the recent in-vitro work by Rindom et al.^47^, the in-vivo results presented herein strengthen the notion that mechanical stimuli per se, uncoupled from the active contraction, is an important signalling cue for protein synthesis, yet specific signalling cues from the excitation are important to achieve a full activation of protein synthesis and following hypertrophy. Further, JNK is likely activated by mechanical stimuli per se, further indicating that this is important for eliciting the mechanically-induced hypertrophic response previously observed with long-term strength exercise^22^. Also, it is likely that both mTOR and p70S6K are part of a mechanotransduction network in conjunction with JNK, modulated both by mechanical stimuli and other exercise-related stimuli^21,54^ like the excitation–contraction coupling and metabolic cues, all contributing to eliciting the hypertrophic response.

### Effects of exercise duration and load on FAK and JNK abundance

The abundance of FAK and JNK, but not mTOR, was affected to a considerable degree by the duration of the exercise experiment per se, which was explained by a reduced abundance in the assessed soluble protein fraction after the 28-min exercise bout in both the control and exercised leg. Stretch increases the phosphorylation of FAK in rat skeletal muscle within 20 seconds^55^, and translocation of activated FAK from sarcomeres to a nuclei-associated, Triton X-100 insoluble, fraction has been reported to occur in the heart within 60 min of pressure overload, corresponding with increases in protein synthesis and myofibrillar assembly^56^. Intriguingly, total tyrosine 397 phosphorylation of FAK has been reported to be decreased in human elbow extensor muscle as a function of time, 15 min and 3 h after resistance exercise, in function of whether the exercise was preceded by a bout of aerobic exercise^57^.

Our present data thus indicate a possible experimental model-related interference with FAK and JNK signalling through effects on their solubility and/or calcium-induced degradation^58^ during the 28-min of exercise. Both FAK and JNK are part of a rescue pathway that can be conditioned by local ischemia to protect skeletal muscle from reperfusion injury^59,60^, suggesting that systemic effects in the exercising and control muscle may be due to the experimental procedure, including, but not limited to, exercise-induced ischemia. In this respect, an altered solubility may have influenced the observed increase in the specific phosphorylation of JNK 28-min, but not 2 and 10-min, after exercise. That the JNK abundance in the assessed soluble fraction was lower in the high-load than the low-load group after the 28-min of exercise thus strengthens our conclusion that JNK is preferentially activated by load, given the notion that it is the activated form that translocates to the insoluble fraction^61^. Probably this relates to the activation of JNK in rat skeletal muscle by stretch or other mechanical cues^33^. However, we cannot rule out a possible exercise- and load-effect also on FAK that we were not able to detect, given the addressed limitations of the experimental setup, related to the rapid activation- and translocation dynamics of FAK^62^, as it has previously been indicated as an early mechanotransducing kinase in skeletal muscle^1,37^.

In conclusion, our study shows that JNK is specifically activated in a load- and exercise duration-dependent manner during in vivo strength exercise. It also furthers our understanding of the muscle-, exercise duration- and load-specific acute responses to electrically paced-exercise for the investigated kinases FAK, mTOR, p70S6K and JNK, and indicates how their regulation is linked to an increased protein synthesis and hypertrophic response.

## Materials and methods

Animals were kept at the animal facility at the Department of Biosciences, University of Oslo, housed with a 12 h light/dark cycle with ad libitum access to food and water. A total of 50 rats were used for the experiments. All animal experiments were conducted in accordance with the Norwegian Animal Welfare Act of 20th December 1974, and all experiments were approved by the Norwegian Animal Research Committee before initiation. The Norwegian Animal Research Authority provided governance to ensure that facilities and experiments were in accordance with the Act, National Regulations of January 15th, 1996, and the European Convention for the Protection of Vertebrate Animals Used for Experimental and Other Scientific Purposes of March 18th, 1986. Animals were euthanized by cervical dislocation while under deep anaesthesia.

### Design

The dorsal flexors, extensor digitorum longus (EDL) and tibialis anterior (TA), of the right lower leg of anesthetized 10 weeks old male Sprague Dawley rats with a mean body weight of 413 g were randomly subjected to electrically paced contractions under a high or low load for 2, 10 or 28 min. The non-stimulated left leg served as a within-animal control. 8–9 animals were used per protocol. Soluble protein from the excised muscles were subjected to electrochemiluminescence (ECL)-based immunoassays to quantify the abundance of FAK, mTOR, p70S6K and JNK, as well as the respective level of phospho-Y397-FAK, phospho-S2448-mTOR, phospho-T421/S424-p70S6K, and phospho-T183/Y185-JNK.

### Electrically paced exercise

Muscle contractions were electrically paced under identical excitation patterns to have the stimulated right lower leg perform controlled isometric (high load) or concentric (low load) tetanic contractions under inhalation anaesthesia (isoflurane) essentially as described before^22^.

The skin of the lower leg of anesthetized rats was shaved, and two platinum skin electrodes measuring 5 times 6 mm covered with electrode gel (Spectra 360; Parker Laboratories) were placed on the skin over the dorsiflexor muscle group (TA and EDL) of the right leg about 5 mm apart. The electrodes were connected to a pulse generator (Pulsar 6 bp-a/s; FHC).

Exercise was conducted over 90, 360, or 900 short tetanic contractions during 2, 10 or 28 min, each contraction lasting about 120 ms. Stimulation was delivered in trains of 10 consecutive 0.5 ms symmetrical bipolar pulses delivered at an instantaneous frequency of 150 Hz at amplitude 30–45 V. Each pulse train was delivered every 600 ms repeated 30 times. For the 2 min protocol, each such series was repeated every 40 s three times. For the 10 min protocol, each series was repeated every 40 s six times, and each such session was delivered every 360 s two times. For the 28 min protocol, each session was repeated five times.

During exercise, the right leg was attached to a footplate connected to a DC motor (Dual lever-arm system 305C-LR connected to a 600A Digital Controller; Aurora Scientific, Aurora, ON, Canada) in length-control mode, allowing the load to be differentiated during contraction. The high-load group performed pure isometric contractions while the low-load group performed mainly high velocity (550°/s) concentric contractions with 50–60% of the peak force produced in the high-load group. The exercise was performed during the rats’ dark cycle, and different groups were always exercised alternately to minimize potential circadian differences in exercise response. The foot was positioned at an angle of 90° relative to the tibia during the contraction in the high-load group while the low-load group moved from 128.5° to 90°. EDL and TA from the stimulated (i.e., exercising) right leg and then the non-stimulated (i.e., control) left leg were harvested in sequential order immediately after the exercise while rats were still anaesthetised, blotted dry from blood, and snap frozen in liquid nitrogen as previously described^22^.

### Protein extraction

Homogenate was prepared from pooled 20-µm-thick cryosections in ice-cold RIPA buffer (~ 3 mm3 volume per 200 µl of 2% Triton X-100, 1% NP-40, 300 mM NaCl, 20 mM Tris base, 2 mM EDTA, 2 mM EGTA; including one PhosStop tablet, and one tablet of Complete-mini EDTA-Free reagent per 10 ml buffer (Roche Diagnostics, Mannheim, Germany)) with the help of a rotor–stator mixer (Polytron PT1200, KINEMATICA. AG, Lucerne, Switzerland). The soluble fraction of proteins was recovered from the supernatant after a 5 min centrifugation at 5′000 g at 4 °C, and protein concentration determined essentially as described previously^37^.

### ECL-based immunoassay

A volume of supernatant corresponding to 33 µg of soluble muscle protein was analysed per sample for the abundance of p70S6K and phospho-T421/S424-p70S6K, mTOR and phospho-S2448-JNK, JNK and phosphor-T183/Y185-JNK, FAK and phospho-Y397-FAK with a validated MESO scale system (K15114D-1, K15170D-1, K15111D-1, or customized U-PLEX development system; Meso Scale Discovery, USA) for electrochemiluminescence-based multiplex measurements in an ELISA plate as described previously^37^.

The abundance of a respective kinase and its phosphorylation site(s) was analysed on the same ELISA plate with one sample per sample point. Samples from TA and EDL muscle of the 28-min of exercise, and TA muscle from the 2 and 10 min of exercise, were analysed in a separate run on different days. Aliquots from four reference samples were run on both ELISA plates that were used to analyse the 28-min and 2 & 10-min sample points, respectively. The signal values (in counts) for the references were used to scale the ECL signals (in counts) for the kinase abundance and phosphorylation from the two runs (plates). This was achieved using the ratio between the average signals over the four reference samples on the respective plate as scaling factor. This procedure was carried out to control for inter-assay variability due to differences in sample handling on the day of the experiment and variability in the quantitative relationship between the commercial and custom-prepared MESO scale plates from different batches.

The values for the resulting ECL signal in counts were exported in Microsoft Excel for Windows 365 (Microsoft Corporation, Redmond, USA) and used to express the relative abundance of each kinase per muscle protein, and calculate the specific phosphorylation of each kinase from the ratio of the ECL signal for the phosphorylated kinase versus the ECL signal for the respective kinase abundance. The fold differences in the specific phosphorylation of a kinase with exercise was calculated from the ratio of the respective values for the specific phosphorylation between muscles from the exercised leg and its contralateral control.

### Statistics

Data analysis and graphing was carried out with Prism version 9.0.0 for macOS (Graphpad Software, LLC). Repeated measures ANOVAs were carried out on the ECL-based values for the kinase abundance and specific phosphorylation for the repeated factors exercise (control left leg, exercised right leg) and where applicable muscle (EDL, TA), and the not-repeated factor load (high load, low load) and duration of exercise (2, 10 or 28 min). Sphericity was assessed with Mauchly's test statistic (this assumption was never rejected).

Main effects (p-values) from the three-way ANOVA for the factors exercise, load and muscle type (corresponding to Figs. 1 and 2), or exercise, load and exercise duration (corresponding to Figs. 3 and 4) are presented in the results section and in Table 1. Post hoc effects were localized with a Tukey’s multiple comparisons test and are denoted in the figures. Significance was declared at p < 0.05. All potential outliers are included in the statistics.

## Supplementary Information

Supplementary Information 1.
