# Supplementary material for: JNK activation in TA and EDL muscle is load-dependent in rats receiving identical excitation patterns
Source: Sci Rep. 2021 Aug 12;11:16405. doi: 10.1038/s41598-021-94930-x (PMC8361015; doi:10.1038/s41598-021-94930-x)
Supplement: Supplementary file 1 — Supplementary Information 1. [file 41598_2021_94930_MOESM1_ESM.pdf]

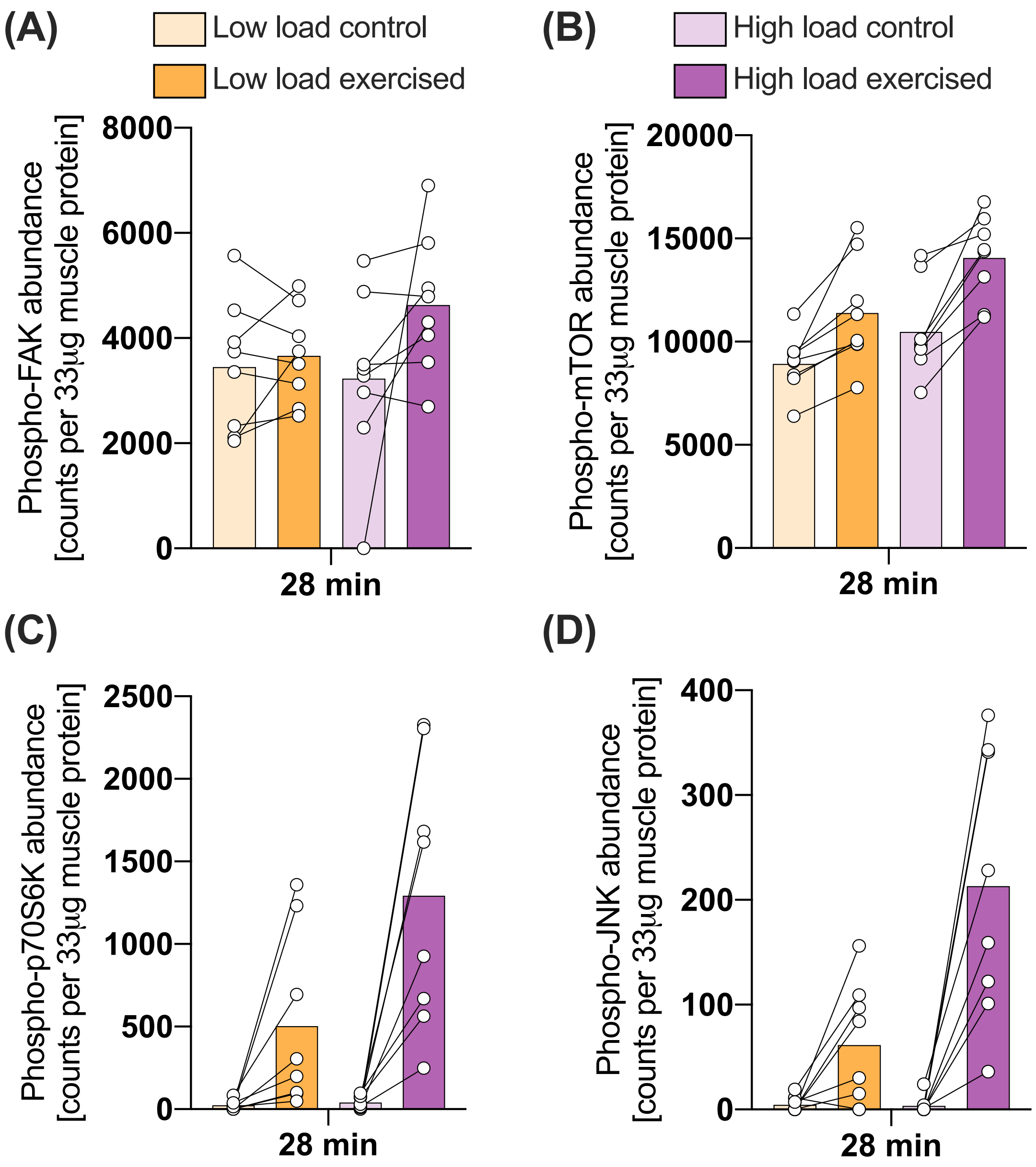

SUPPL. FIGURE 1 Abundance of phosphorylated protein (total count) of Y397-FAK (A), pS2448-mTOR (B), pT421/S424-p70S6K (C), and pT183/Y185-JNK (D) as measured in 33 microgram soluble protein from exercised and contralateral control EDL muscles after a 28-minute exercise stimulus with a high or a low load. Data are presented as individual muscle values (circles), contralateral comparisons (lines) and group means (bars) (n=8 per group).

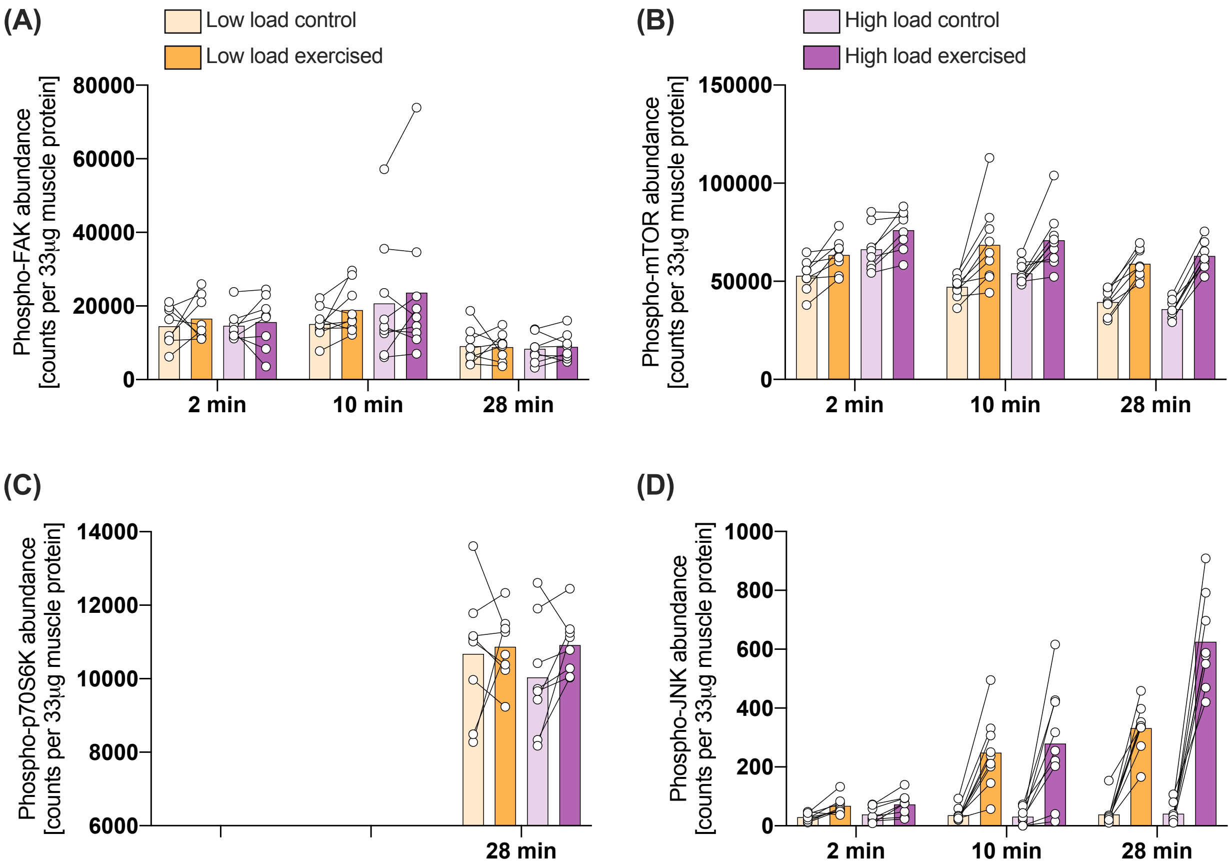

SUPPL. FIGURE 2 Abundance of phosphorylated protein (total count) of Y397-FAK (A), pS2448-mTOR (B), pT421/S424-p70S6K (C), and pT183/Y185-JNK (D) as measured in 33 microgram soluble protein from exercised and contralateral control TA muscles after a 2-, 10- or 28-minute exercise stimulus with a high or a low load. Data are presented as individual muscle values (circles), contralateral comparisons (lines) and group means (bars) (n=8 per group for the 2 min and 28 min protocol, n=9 per group for the 10 min protocol).
